# Spontaneous Spinal Epidural Hematoma in a Patient on Apixaban for Nonvalvular Atrial Fibrillation

**DOI:** 10.1155/2020/7419050

**Published:** 2020-04-14

**Authors:** Ahmad El Alayli, Logeswari Neelakandan, Hicham Krayem

**Affiliations:** Detroit Medical Center Sinai Grace Hospital, Detroit, MI, USA

## Abstract

**Background:**

With the rise in the use of direct oral anticoagulants (DOACs), more hemorrhagic complications are being encountered. Since the first description of a case of spontaneous spinal epidural hematoma (SSEH) related to the utilization of DOACs in 2012, there have been few reports describing a similar association. However, no cases so far have reported an association between SSEHs and apixaban. *Case Description*: A 76-year-old lady, with a history of nonvalvular atrial fibrillation, presented with a new onset of progressive left lower and upper extremity weakness. She reported back pain and numbness in the left leg up to the knee along with numbness in the left arm up to the shoulder. A CT scan of the neck was suggestive of an epidural hematoma extending from C2-C3 level to C6-C7. As the patient was on apixaban at the time, surgical treatment was delayed for two days to decrease the risk of intraoperative bleeding. Nine days later, she was discharged. Her physical exam was almost unchanged from that on presentation, except for resolution of pain and minimal improvement in motor power in her left lower extremity from 1/5 to 2/5 distally.

**Conclusions:**

Spinal hematomas represent surgical emergencies with earlier intervention portending better outcome. Based on the few case reports that point to DOACs as a potential culprit, it appears that a high suspicion index resulting in earlier SSEH diagnosis and intervention is crucial for improved neurological outcome and recovery. Prompt diagnosis remains a challenge, especially that SSEH can mimic cerebrovascular accidents.

## 1. Background

Since the first description of a case of SH associated with the use of a DOAC (rivaroxaban) in 2012 [1], there have been a total of 10 other reports describing both SSEH and spinal subdural hematomas (SSDHs) in association with different DOACs [2–9]. As the use of DOACs continues to increase [10], more similar cases are expected to complicate treatment with these agents. SSEH represents the vast majority of cases of SH [11]. However, no cases so far have been reported about an association between apixaban and SSEH (Table 1). Despite reports of successful medical management [13, 14], surgical decompression remains the cornerstone of management of these patients with earlier intervention correlating with better outcome [15]. However, even with the recent FDA approval of idarucizumab as a reversal agent for dabigatran and andexanet alfa for apixaban and rivaroxaban [16], the limited availability and experience with these agents complicates the timing of surgery. We hope that this case report and literature review will help in establishing a better idea about the optimal management of these patients and raise awareness about the importance of having a high index of suspicion for this condition when encountering a patient on DOACs.

## 2. Case Report

A 76-year-old African American lady presented to the hospital with new onset weakness and back pain. Her medical history consisted of essential hypertension, atrial fibrillation, abdominal aortic aneurysm, sick sinus syndrome, asthma, and gout. She reported that her back pain started after she came back from the bathroom at 4 : 00 a.m. while trying to sit down on the bed. The patient described the pain as shooting in nature, severe in intensity, and radiating down her left arm with no clear exacerbating or relieving factors. Over the following few minutes, she developed progressive weakness in her left lower and upper extremities and later on became unable to move her left leg which led to her being transferred to the Medical Intensive Care Unit. She also developed numbness in her left leg up to the level of the knee and in her left arm up to the level of the shoulder. There was no history of any urinary or stool incontinence or retention or any previous similar episodes.

The patient had been recently diagnosed with atrial fibrillation one month prior to presentation in an outside hospital and had since been maintained on apixaban 1 tablet 5 mg twice daily. Her other home medications included alprazolam, allopurinol, losartan, hydrochlorothiazide, and metoprolol. She reported full compliance with her medications prior to presentation. A dual chamber pacemaker was inserted 11 days before this visit in an outside hospital, and her other surgical history included a hysterectomy several years ago for uterine fibroids. She denied any history of smoking, alcohol abuse, or illicit substances usage. The patient did not have previous bleeding history.

The patient's vital signs on presentation were within normal limits, and her neurological exam showed a patient alert and oriented to time, place, and person with intact speech. Her motor power was 5/5 in her right upper extremity proximally and 4/5 distally and 3/5 in her left upper extremity proximally and 1/5 distally. In her lower extremities, the motor power was 5/5 on the right and 1/5 all over on the left. She had decreased sensation to light touch in her left upper and lower extremities. She had no clonus, and Hoffman and Babinski signs were negative bilaterally. Her reflexes were 2+ all over and symmetric.

Initial lab tests were significant for hypokalemia (3.1 mmol/L), leukocytosis (12700 WBCs per microliter), and slight anemia with a hemoglobin level of 9.3 g/dl with unknown baseline. Her other lab tests including liver function and renal function were within normal limits.

**Figure 1:**
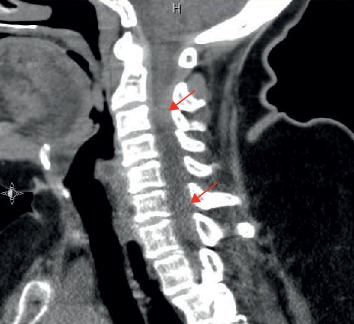
Sagittal CT scan image showing posterior epidural hematoma.

Given her presentation of acute focal neurological deficit, a noncontrast head CT scan was ordered and showed signs of chronic vascular ischemic disease only. The patient continued to have severe back pain, and having a history of abdominal aortic aneurysm, an acute aortic dissection was suspected, and hence, a CT angiogram of the abdomen, pelvis, and thorax was ordered. The CT angiogram did not show any signs of aortic dissection. A CT scan of the neck was performed to investigate the cause of the left upper extremity weakness and numbness. It showed an epidural soft tissue density extending along the left aspect of the spinal canal causing compression and marked rightward deviation of the cord concerning for an epidural hematoma extending from C2-C3 level to C6-C7 (Figure 1).

As the patient was on apixaban at the time, a decision was made to delay surgical treatment for 2 days to decrease the risk of intraoperative bleeding. A C2-C7 laminectomy, hematoma exclusion, and spinal fusion were then performed. On discharge, 9 days later, her physical exam was mostly unchanged from that on presentation except for the resolution of pain and improvement in motor power in her left lower extremity from 1/5 to 2/5 distally.

## 3. Discussion and Conclusions

Spinal hematomas represent surgical emergencies with earlier intervention portending better outcome [15]. The acute presentation, however, represents an important diagnostic challenge [15] especially as a mimicker of cerebrovascular accidents [18]. Even though no direct trauma is involved in spontaneous spinal hematomas, some activities have been associated with onset such as stretch exercises [19]and Valsalva maneuvers during trumpet playing [20] and defecation [3], for example, which might have been the case in our patient as well. Even though new reversal agents have been recently approved for dabigatran, apixaban, and rivaroxaban [16], the lack of their widespread use makes it essential to identify the best evidence-supported choice for surgical timing of evacuation. Based on the few case reports that have been published so far, it seems that the benefit of earlier intervention outweighs the risk of surgical bleed.

According to our literature review, 4 patients underwent surgical intervention within 24 hours of symptoms onset, and 3 of these 4 patients (75%) had excellent outcomes with unknown outcome in the third one [2, 4, 5]. Preceding the emergent surgical interventions, prothrombin complex concentrate was given to one patient and fresh frozen plasma to the other which has been shown in some previous studies to be an effective reversal method for factor *X* inhibitors [21]. There was no associated bleeding with either surgical intervention. On the other hand, 5 cases (including this report) had delayed surgical intervention (>24 hours) [5–9]. Among these cases, only 2 had good recovery (40%), and 3 had bad outcomes. No perioperative bleed was reported in any of the cases. The short follow-up period in most of these articles including ours (9 days) represent a major limiting factor in most of these reports.

The number of cases reported is insufficient to reach any statistically significant result, but seems to be pointing towards the importance of early surgical intervention for neurologic recovery. With the development of new reversal agents and increased experience with the current ones, earlier surgical intervention is most likely to increase in frequency as surgeons become more comfortable operating on these patients leading hopefully to less neurologic deficit. This means that a high suspicion index leading to earlier diagnosis is key for timely intervention, and we hope that this article will help in raising awareness about this condition as a potential complication of DOACs use, especially with the significant and continuous increase in their utilization.

## Figures and Tables

**Table 1:** Cases of SSDH and SSEH associated with the use of DOACs described in the literature.

Study	Age of the patient (Years), gender	Presenting symptoms	DOAC used	Site (epidural/subdural)	Radiographic findings	Intervention used	Time from last DOAC dose to procedure (>24 hours)	Onset of neurologic deficit symptoms to intervention (>24 hours)	Reversal agent used	Recovery/marked improvement
This case	76, F	Severe back pain and progressing LUE and LLE weakness and numbness	Apixaban	Epidural	CT: epidural hematoma from C2-C3 to C6-C7	C2–C7 laminectomy and hematoma evacuation	Yes	Yes	None	No
Jaeger et al. [1]	61, F	Severe back pain with bilateral LE weakness and numbness	Rivaroxaban	Epidural	MRI: epidural hematoma C2–T8	None	N/A	N/A	None	Yes
Bamps et al. [2]	70, M	Severe cervical pain, tetraplegia, sensory loss, and severe autonomic instability	Dabigatran	Epidural	CT: C2–C4 epidural hematoma	C2–C4 laminectomy and hematoma evacuation	No	No	Prothrombin complex concentrate	Yes
Ozel et al. [3]	69, F	Severe cervical pain followed by quadriplegia	Rivaroxaban	Epidural	CT: C2–C4 epidural hematoma	None	N/A	N/A	None	Yes
Ismail et al. [4]	72, M	Severe back pain, bilateral lower extremities weakness and numbness, and urinary incontinence	Rivaroxaban	Epidural	MRI: T11-L2 epidural hematoma	T11–L2 laminectomy and hematoma evacuation	No	No	Fresh frozen plasma	Yes
Goldfine et al. [5]	74, M	Gradual onset neck pain and waxing and waning paralysis of all extremities with fecal incontinence	Rivaroxaban	Epidural	MRI: foramen magnum-C7 epidural hematoma	Surgery and hematoma evacuation	Yes	No	None	U/K
Zaarour et al. [6]	58, M	Sudden back pain with progressive numbness in bilateral lower extremities followed by progressive weakness	Rivaroxaban	Subdural	MRI: C7-T2 subdural hematoma with edema	Steroids followed by laminectomy and evacuation	Yes	Yes	Aminocaproic acid	Yes
Castillo et al. [7]	69, M	Excruciating back pain with progressing lower extremities weakness leading to paraplegia and bowel and bladder dysfunction	Rivaroxaban	Subdural	MRI: T3-Conus medullaris subdural hematoma	Cervical and lumbar CSF drains	Yes	Yes	None	No
Dargazanli et al. [8]	72, M	Back pain, flaccid paraplegia, positive Babinski, thermalogic hypoesthesia, absent knee and ankle reflexes, and decreased anal tone	Rivaroxaban	Subdural	MRI: T6–T8 subdural hematoma	T6–T8 laminectomy with hematoma evacuation, received IV steroids after surgery	Yes	Yes	Prothrombin complex concentrate	No
Colell et al. [9]	75, F	Paraparesis, hyperreflexia, hypoesthesia, no plantar reflex on the left	Dabigatran switched to apixaban before episode	Subdural	MRI: large subdural hemorrhage with discontinuous cervical-dorsal-lumbosacral involvement with secondary spinal cord compression	D1–D3 vertebral laminectomy with hematoma evacuation followed by D4–D7 laminectomy with hematoma evacuation 1 week later	Yes	Yes	None	Yes
Radcliff et al. [12]	53, F	Pain in the right buttock and leg, numbness in bilateral buttock and sensation of incomplete bladder emptying	Rivaroxaban	Epidural	MRI: spinal epidural hematoma at right L4-L5	L4-L5 laminectomy and evacuation of hematoma	U/K	No	None	Yes

## Abbreviations

DOACs: Direct oral anticoagulants
SH: Spinal hematoma
SSEH: Spontaneous spinal epidural hematoma
SSDH: Spinal subdural hematoma.
